# Characteristics and outcome of critically ill patients with coronavirus disease-2019 (COVID-19) pneumonia admitted to a tertiary care center in the United Arab Emirates during the first wave of the SARS-CoV-2 pandemic. A retrospective analysis

**DOI:** 10.1371/journal.pone.0251687

**Published:** 2021-10-22

**Authors:** Khaled Ismail, Hatim Bensasi, Ahmed Taha, Aamir Nazir, Mohamed Abdelkhalek, Walid Mohamed, Dipak Lodhe, Samuel Buschbeck, Michael Bauer, Yasser Sakr

**Affiliations:** 1 Department of Anesthesiology and Critical Care Medicine, Al Ain Hospital, SEHA, Abu Dhabi, UAE; 2 Department of Critical Care, Cleveland Clinic, Abu Dhabi, UAE; 3 Department of Anesthesiology and Intensive Care Medicine, Jena University Hospital, Jena, Germany; Heidelberg University Hospital, GERMANY

## Abstract

**Background:**

The aim of this study was to describe the clinical characteristics and outcome of patients with coronavirus disease-2019 (COVID-19) pneumonia admitted to an intensive care unit (ICU) of a tertiary care center in the United Arab Emirates (UAE) and to identify early risk factors for in-hospital mortality in these patients.

**Methods:**

A total of 371 adult patients (>18 years) admitted to the ICU of Al Ain Hospital between March 16 and July 19, 2020 with SARS-CoV-2 infection confirmed using real-time reverse transcription polymerase chain reaction (rt-PCR) on nasopharyngeal swabs were included.

**Results:**

The mean patient age was 53 years (standard deviation = 13). Patients were mostly male (n = 314 [84.6%]) and of South Asian origin (n = 231 [62.3%]). Invasive mechanical ventilation was required in 182 (49.1%) patients for a median of 11 days (25–75% interquartile range: 6–17). During the ICU stay, renal replacement therapy was required in 87 (23.5%) and vasopressor therapy in 190 (51.2%) patients. ICU and hospital lengths of stay were 9 (IQ: 5–17) and 18 (IQ: 13–29) days, respectively and ICU and hospital mortality rates were both 20.2%. In a multivariable analysis with in-hospital mortality as the dependent variable, greater Acute Physiology and Chronic Health Evaluation II score on ICU admission, diarrhea prior to hospital admission, greater, admission from hospital ward, and higher lactate dehydrogenase levels and neutrophil:lymphocyte ratio on admission to the ICU were independently associated with higher risk of in-hospital mortality.

**Conclusion:**

In this cohort of patients admitted to the ICU of a tertiary hospital in the UAE, COVID-19 pneumonia was associated with high morbidity and mortality rates. Identifying patients at high risk of death may help detect future therapeutic targets.

## Introduction

As the health burden from coronavirus disease-2019 (COVID-19) due to the novel SARS-CoV-2 continues to rise, the world’s governments, institutions, and agencies are still working toward understanding who is most at risk of severe complications and death [1]. The subset of patients who require admission to the intensive care unit (ICU) has been reported to be at particular risk of developing major complications with a high mortality rate [2–20]. The characteristics and outcomes of these patients have been reported in several studies from China [10, 21–24], Europe [2, 3, 5, 9, 11, 14, 17–20], and North America [4, 6, 7, 25]; however, similar data from other geographic regions are scarce. Such data are important to understand the disease burden worldwide. Indeed, the COVID-19 epidemic has affected countries around the globe to different extents. Notably, most of the large published ICU cohorts have included patients from countries that were overwhelmed during the early months of the epidemic and may not be representative of critically ill patients with severe SARS-CoV-2 infections worldwide [2–7, 9–11, 14, 17, 18, 21–25].

The United Arab Emirates (UAE) is known for its diverse population with hundreds of nationalities within its borders, creating considerable challenges in terms of cultural diversity. Nonetheless, access to healthcare services is regarded as adequate and healthcare infrastructure is considered better than in other Middle Eastern countries. The UAE has experienced relatively low COVID-19 mortality per capita [26]; its experience may provide valuable insight for other countries to prepare for future waves of the pandemic.

The aim of this study was, therefore, to describe the clinical characteristics and outcomes of critically ill patients with COVID-19 pneumonia admitted to a tertiary care center in the UAE during the first wave of the pandemic. We also evaluated the risk factors for in-hospital mortality that may help is early risk stratification of these patients. Our hypothesis was that severe COVID-19 would be associated with high morbidity and mortality rates in the ICU and that parameters of tissue inflammation on admission to the ICU would play an important role in determining the COVID-19-associated risk of death.

## Methods

The study was approved by the institutional review board of the Department of Health, Abu Dhabi (PO box 5674, Abu Dhabi, UAE, application number: DOH/CVDC/ 2020/1669), which waived informed consent due to the retrospective nature of data collection. Source data were accessed by the attending physicians (KI and HB). Data were anonymously recorded prior to further handling and analysis. All adult patients (>18 years) admitted to the ICU of Al Ain Hospital between March 16 and July 19, 2020, with SARS-CoV-2 infection confirmed using real-time reverse transcription polymerase chain reaction (rt-PCR) on nasopharyngeal swabs and radiologic evidence of respiratory infections were considered for inclusion in the study. Patients with incomplete records were excluded from the analysis. Follow up was completed until death or hospital discharge, whichever occurred first. Preliminary results on the potential impact of camostat mesylate therapy on outcome in this cohort were recently published [27].

Patient records were reviewed by the attending physicians (HB and KI). Demographic data, referring facility, preexisting comorbid conditions, and the initial manifestations of COVID-19 prior to hospital admission were recorded. Laboratory parameters and therapeutic interventions on admission to the ICU were retrieved electronically from the local patient data management system (Cerner corp, North Kansas City, Missouri, USA) and recorded. The Acute Physiology and Chronic Health Evaluation II (APACHE II) score was calculated from the data obtained within 24 hours of admission to the ICU [28]. Data were then anonymously recorded for further handling and analysis.

### Standard of care

Al Ain Hospital is a tertiary care hospital located in Abu Dhabi, the capital of the UAE, which was devoted to the isolation and treatment of patients with suspected or confirmed SARS-CoV-2 infections during the study period. The ICU of Al Ain Hospital is an interdisciplinary ICU with a normal capacity of 23 beds. During the study period, the capacity of the ICU was extended to 96 beds, fully equipped with advanced organ support therapies, including, but not limited to, invasive mechanical ventilation and renal replacement therapy. Intensivists were available 24-h/day with a background in anesthesiology and intensive care and qualified according to local regulations. The medical staff, including attending physicians, nursing staff, and physiotherapists, performed daily patient rounds. Standard ICU management followed the Standard Operating Procedures of the Al Ain Hospital and was in accordance with international guidelines [29]. Patients were isolated in single rooms and received medical care with a 1:1 nurse:patient ratio. Infection control measures were implemented and monitored by the department of infection control of Al Ain Hospital. Admission to the ICU was at the discretion of the attending physician. Antiviral and adjunctive therapies were prescribed at the discretion of the attending physician. Invasive mechanical ventilation was performed using lung protective ventilation settings [30].

### Outcome parameters

The primary outcome parameter was in-hospital mortality. Secondary outcome parameters included death in the ICU, ICU and hospital lengths of stay, and use of mechanical ventilation, renal replacement therapy or vasopressor therapy during the ICU stay.

### Statistical analysis

All data were processed and analyzed in Al Ain Hospital, in collaboration with Jena University Hospital, Jena, Germany. Data were analyzed using IBM^®^ SPSS^®^ Statistics software, v.21 for Windows (IBM, Somers, NY, USA). Data are summarized using means with standard deviation, medians and interquartile ranges (IQ), or numbers and percentages. Difference testing between groups was performed using Student’s t test, Mann–Whitney test, Chi–square test or Fisher’s exact test, as appropriate. The Kolmogorov–Smirnov test was used to verify whether there were significant deviations from the normality assumption of continuous variables.

To determine independent risk factors for in-hospital death, we performed a multivariable logistic regression analysis with in-hospital death as the dependent variable. Covariates to be included in the final model were based on a univariate logistic regression analysis (p<0.2) of demographic variables (age, sex, and ethnicity), referring facility, initial symptoms prior to ICU admission, comorbid conditions, need for mechanical ventilation on admission to the ICU, inflammatory parameters, D-dimer levels, laboratory parameters of organ function, and complete blood count blood picture (the neutrophil:lymphocyte ratio [NLR] was included instead of the individual counts) on admission to the ICU. Colinearity between variables was ruled out before covariates were introduced in the model. Goodness of fit was tested using a Hosmer and Lemeshow test, and odds ratios (OR) with 95% confidence intervals (CIs) were computed. To reduce the number of variables included in the multivariable model, a forward stepwise approach was used with an inclusion criteria of p<0.2 and exclusion at p>0.1. Secondary outcome parameters were assessed using descriptive statistics and compared between survivors and non-survivors.

Receiver operator characteristic (ROC) curves and the areas under the curves (AUC) were computed to determine the value of the independent risk factors for discriminating between survivors and non-survivors. The best cut-off point was defined using the Youdin index, and sensitivity, specificity, negative predictive value (NPV), and positive predictive value (PPV) were calculated.

All reported p values are two-sided and a p value <0.05 was considered to indicate statistical significance.

## Results

### Characteristics of the study group

**Fig 1:**
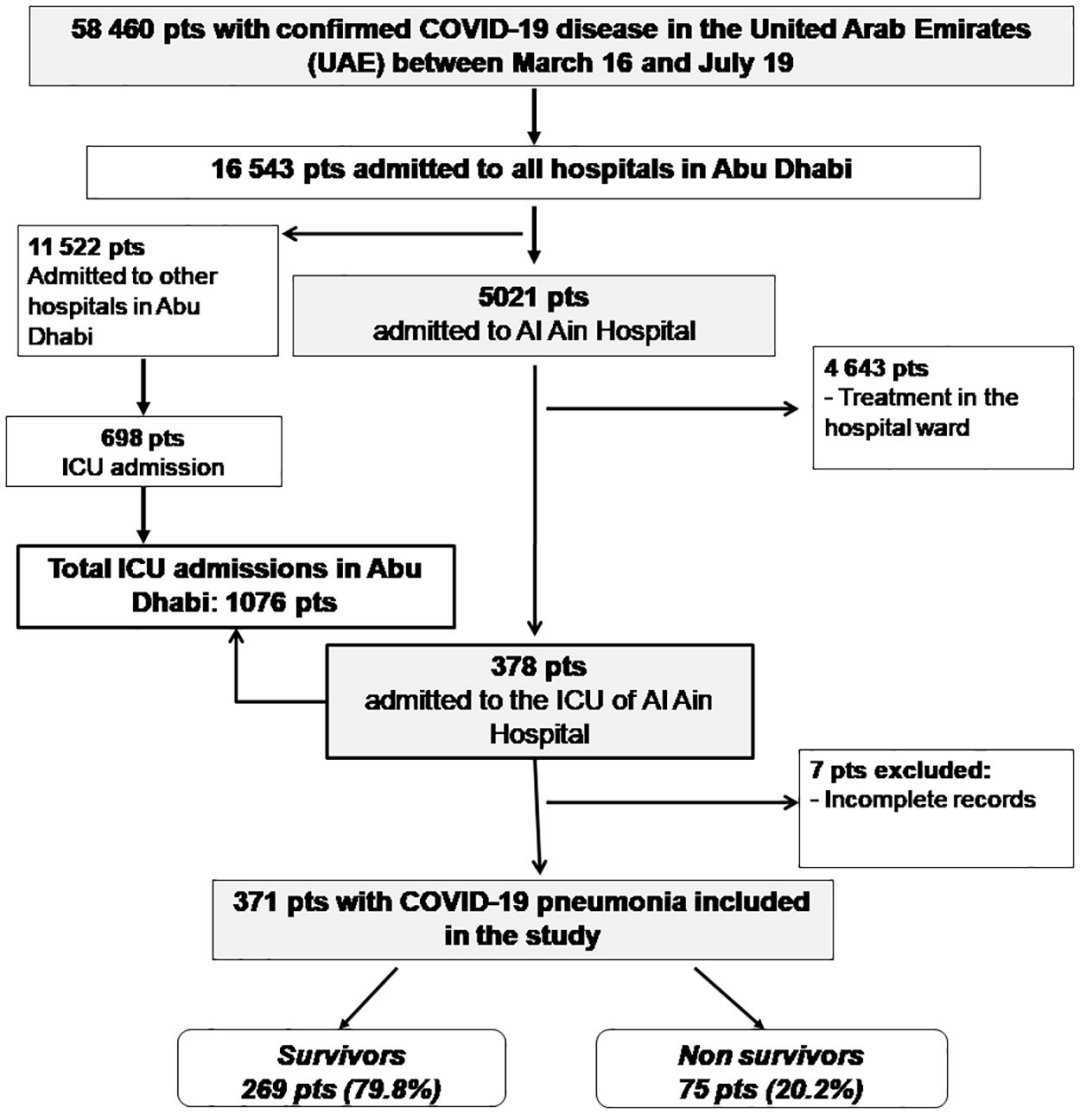
Study inclusion. Flow diagram representing inclusion in the study.

During the study period, 5021 patients were admitted to Al Ain Hospital with confirmed SARS-CoV-2 infection. Of 378 patients who required admission to the ICU, 371 patients had documented COVID-19 pneunonia and constituted the study group (Fig 1). Seven patients were excluded due to incomplete records, in which the diagnosis of pneumonia was not established. The characteristics of the study group are presented in Table 1. The mean age was 53 years (SD = 13); the majority were male (84.6%) and of south Asian origin (62.3%). The most common comorbid conditions were diabetes mellitus (44.2%), systemic hypertension (43.1%), and cardiovascular disease (17.0). About half of the patients (52%) were referred from another hospital, and 40% were admitted to the ICU directly from the emergency department of our hospital. The symptoms most commonly reported prior to hospital admission were cough (79.8%), fever (77.6%), dyspnea (77.4%), malaise (49.6%), and headache (32.1%). South Asian patients were more commonly younger males, had less comorbid, had lower APACHE II score on admission to the ICU, and greater BMI than Arabs (S1 Table).

**Table 1:** Characteristics of the study group on admission to the ICU.

	All patients	Survivors	Non-survivors	p value
N	371	269	75	
Age, years, mean ±SD	53±13	52±14	60±14	<0.001
Male (%)	314 (84.6)	252 (85.1)	62 (82.7)	0.596
Weight, kg, mean ±SD	80.8±19.6	80.7±19.7	80.9±19.3	0.457
BMI, kg/m^2^, mean ±SD	31.5±23.2	32.0± 25.8	29.6± 6.4	0.281
Referring facility, n (%)				0.027
Other hospital, same city	167 (45.0)	133 (44.9)	34 (45.3)	
Primary admission	149 (40.2)	127 (42.9)	22 (29.3)	
Hospital ward	30 (8.1)	19 (6.4)	11 (14.7)	
Other hospital, another city	25 (6.7)	17 (5.7)	8 (10.7)	
Ethnicity, n (%)				0.104
South Asian	231 (62.3)	190 (64.2)	41 (54.7)	
Arab	116 (31.3)	83 (30.9)	33 (44.0)	
Asian, others	19 (5.1)	18 (6.7)	1 (1.3)	
Other	5 (1.3)	5 (1.7)	0	
Comorbid conditions, n (%)				
Diabetes mellitus	164 (44.2)	125 (42.2)	39 (52.0)	0.128
Systemic hypertension	160 (43.1)	125 (42.2)	35 (46.7)	0.488
Cardiovascular disease, any	63 (17.0)	42 (14.2)	21 (28.0)	0.004
Ischemic heart diease	40 (10.8)	27 (9.1)	13 (17.3)	0.041
Congestive heart failure	12 (3.2)	8 (2.7)	4 (5.3)	0.272
Atrial fibrillation/flutter	7 (1.9)	5 (1.7)	2 (2.7)	0.633
Heart block	3 (0.8)	2 (0.7)	1 (1.3)	0.493
Valvular heart disease	2 (0.5)	1 (0.3)	1 (1.3)	0.364
Peripheral vasucular disease	1 (0.3)	1 (0.3)	0	1.000
Chronic renal disease, any	50 (13.5)	28 (9.5)	22 (29.3)	<0.001
End stage renal disease	7 (1.9)	4 (1.4)	3 (4.0)	0.150
APACHE II score, mean ±SD	11±8	9±6	18±10	<0.001
Initial symptoms, n (%)				
Cough	296 (79.8)	241 (81.4)	55 (73.3)	0.119
Fever	288 (77.6)	234 (79.1)	54 (72.0)	0.190
Dyspnea	287 (77.4)	231 (78.0)	56 (74.7)	0.533
Malaise	184 (49.6)	155 (52.4)	29 (38.7)	0.034
Headache	119 (32.1)	103 (34.8)	16 (21.3)	0.026
Nausea/vomiting	25 (6.7)	21 (7.1)	4 (5.3)	0.797
Diarrhea	22 (5.9)	14 (4.7)	8 (10.7)	0.052
Productive cough	25 (6.7)	17 (5.7)	8 (10.7)	0.129
Wheeze	2 (0.5)	2 (0.7)	-	1.000

APACHE II: Acute physiologic and chronic health evaluation score, BMI: body mass index, ICU: intensive care unit, SD: standard deviation. Missing values: weight: 3 (2;1), BMI:3 (2;1), APACHE II: 10 (9;1).

### Diagnostic procedures, therapeutic interventions, and antimicrobial therapy

All patients had pulmonary infiltrates in chest x ray examinations. Lung computed tomography (CT) scan was performed in 262 patients and revealed bilateral peripheral ground glass opacities in 254 (96.9%) patients. On the day of admission to the ICU, 54 patients (14.5%) required invasive mechanical ventilation and 21 patients (5.7%) required renal replacement therapy. Anticoagulation using therapeutic dose of enoxaparin sodium (1mg/kg bid) was given in 318 (85.7%) patients; prophylactic anticoagulation (enoxaparin sodium at 0.4 IU/d) was given in 53 (14.3%) patients. Favipiravir was used in 91.6% of patients (n = 340) and camostat in 38% (n = 141). Immunomodulatory therapy using tocilizumab was administered in 143 (38.5%) patients and steroids were given in 62 (16.7%) patients (dexamethasone in 41 (11.1%) patients). Additional antibiotic therapy was given in 270 (72.8%) patients: piperacillin and tazobactam in 124 (33.4%), and doxycycline in 106 (28.6%) patients. Hydroxychloroquine was given to 227 (61.2%) patients.

### Morbidity and mortality

During the ICU stay, renal replacement therapy was required in 87 (23.5%) patients and vasopressor therapy in 190 (51.2%). Invasive mechanical ventilation was required in 182 (49.1%) patients for a median of 11 days (IQ: 6–17). The ICU and hospital lengths of stay were 9 (IQ: 5–17) and 18 (IQ: 13–29) days, respectively, and ICU and hospital mortality rates were both 20.2% (n = 75).

Hospital non-survivors were older, more commonly referred from the hospital ward and other hospitals, more likely to have ischemic heart disease and chronic renal disease, less likely to complain of malaise and headache as the initial symptoms of COVID 19 disease, and had greater APACHE II score than survivors (Table 1). On admission to the ICU, inflammatory parameters, D-dimer levels, direct bilirubin, serum creatinine, urea, and alkaline phosphatase concentrations, neutrophil count, and NLR were higher in non-survivors than in survivors, whereas serum albumin, red blood cell count, hemoglobin, hematocrit, and lymphocyte count were lower in non-survivors than in survivors (Table 2). The ICU length of stay was longer (12 (6–23) vs. 8 (5–16), days, p = 0.035) and hospital length of stay shorter (14 (7–24) vs. 18 (13–29), days, p = 0.001) in non-survivors than in survivors. Non-survivors more commonly required invasive mechanical ventilation (98.7 vs. 37.8%, p<0.001), renal replacement therapy (57.3 vs. 14.9%, p<0.001), and vasopressor therapy (98.7 vs. 39.2%, p<0.001) than did survivors.

**Table 2:** Laboratory parameters on admission to the ICU according to hospital outcome.

	Survivors	Non-survivors	p-value
N	269	75	
Inflammatory parameters			
White blood cell count, x10^9^/L	7.6 (5.7–4.5)	8.8 (6.5–13.7)	0.011
C-reactive protein, mg/L	123 (57–176)	155 (93–243)	0.003
Procalcitonin, ng/ml	0.21 (0.12–0.55)	0.56 (0.28–2.5)	<0.001
Lactate dehydrogenase, IU/L	410 (315–508)	493 (397–633)	<0.001
Ferritin, ng/mL	926 (565–1645)	1127 (710–2745)	0.005
D-dimer, mg/L	0.9 (0.5–1.6)	1.7 (0.8–3.3)	<0.001
Organ function parameters			
Bilirubin, total, μmol/L	9 (6.6–12.3)	9.7 (6.4–13.4)	0.338
Bilirubin, direct, μmol/L	4.4 (3.2–6.4)	5.6 (3.6–9.8)	0.001
Creatinine, μmol/L	79 (67–94)	99 (72–229)	<0.001
Urea, mmol/L	5 (3.6–6.7)	7.5 (4.6–15.3)	<0.001
Serum albumin, g/L	29 (26–33)	26 (22–29)	<0.001
Alkaline phosphatase, IU/L	72 (57–95)	92 (63–126)	0.001
ALT, IU/L	34 (24–55)	28 (20–50)	0.236
AST, IU/L	41 (30–62)	45 (32–63)	0.588
Blood picture			
Red blood cell count, x10^12^/L	4.6 (4.1–5.1)	4.3 (3.8–4.9)	0.046
Platelet count, x10^9^/L	213 (171–287)	207 (152–242)	0.035
Hemoglobin level g/l	130 (116–141)	120 (104–136)	0.012
Hematocrit, %	38 (35–42)	36 (31–40)	0.015
Lymphocyte count, x10^9^/L	1 (0.8–1.3)	0.8 (0.5–1.1)	0.001
Neutrophil count, x10^9^/L	5.9 (4.2–8.8)	7.3 (4.9–12.6)	0.002
Neutrophil:lymphocyte ratio	6.0 (4.1–9.4)	11.9 (5.4–17.4)	<0.001

Data presented as median (interquartile range).

ALT: alanine aminotransferase, AST: aspartate transaminase, IU: international unit.

Missing values (survivors/non-survivors): white blood cell count:7/1, procalcitonin:0/1, LDH:2/1, ferritin:0/1, D-dimer:1/4, bilirubin-direct:7/0, bilirubin-total:4/0, creatinine:0/1, urea:0/1, serum albumin:3/1, alkaline phosphatase: 6/0, ALT:4/0, AST:5/0, red cell count: 7/1, platelet count: 8/2, hemoglobin: 2/0, lymphocyte count: 7/1, neutrophil count: 7/1.

### Risk factors for in-hospital mortality

In a multivariable analysis with in-hospital mortality as the dependent variable, admission from hospital ward (OR: 3.55, 95% CI: 1.26–10.03, p = 0.017), greater APACHE II score on ICU admission (OR:1.13, 95% CI: 1.08–1.18, p<0.001), diarrhea prior to hospital admission (OR: 3.71, 95% CI: 1.10–12.57, p = 0.017), higher NLR (OR: 1.04, 95% CI: 1.01–1.18, p = 0.017), and higher LDH (OR: 1.01, 95% CI: 1.01–1.02, p = 0.026) were independently associated with a higher risk of in-hospital mortality (Table 3 and S3A–S3C Table).

**Table 3:** Summary of logistic regression analysis with in-hospital death as the dependent variable*.

	OR (95% CI)	p value
APACHE II (per point)	1.13 (1.08–1.18)	<0.001
Source of admission		
• Primary admission	R	NA
• Other hospital, same city	1.56 (0.47–5.16)	0.465
• Other hospital, anothercity	0.76 (0.36–1.64)	0.490
• Hospital ward	3.55 (1.26–10.03)	0.017
Initial symptoms		
• Diarrhea	3.71 (1.10–12.57)	0.035
Laboratory parameters on ICU admission		
• Neutrophil:lymphocyte ratio	1.04 (1.01–1.18)	0.017
• Lactate dehydrogenase (per IU/L)	1.01 (1.01–1.02)	0.026

APACHE: acute physiology and chronic health evaluation, CI: Confidence interval, ICU: intensive care unit, OR: odds ratio.

*Forward stepwise approach, excluding 8 patients with missing values.

The presented values are those resulting from the last step of the respective models. Covariate inclusion was based on a univariate logistic regression analysis (p<0.2) within the categories demographic variables (age, sex, and ethnicity), comorbid conditions, the need for mechanical ventilation on admission to the ICU, the initial symptoms prior to ICU admission, inflammatory parameters, D-dimer levels, and laboratory parameters of organ function on admission to the ICU. The neutrophil:lymphocyte ratio was included instead of the individual counts. Hosmer & Lemeshow goodness of fit Chi square: 7.36, p = 0.460.

**Fig 2:**
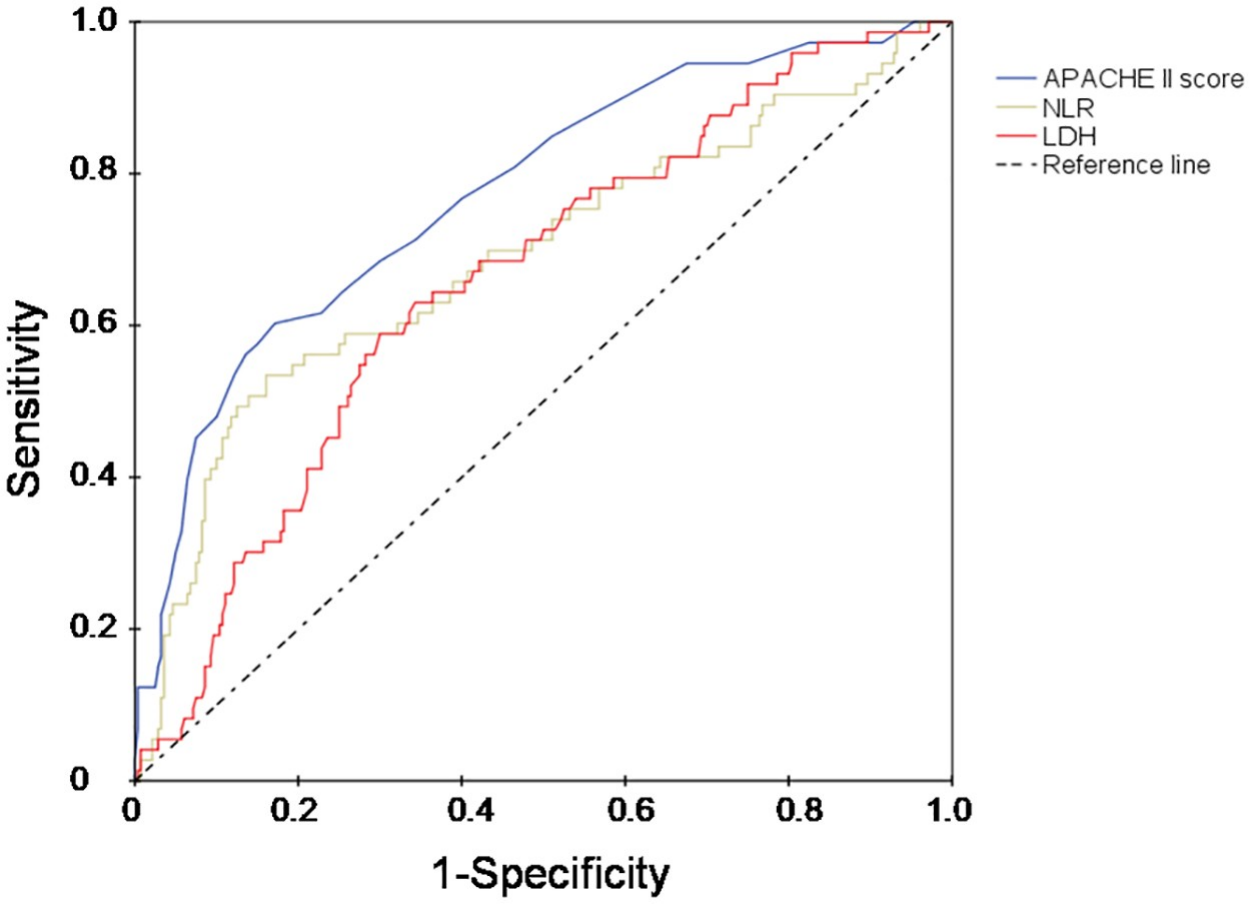
Receiver operator characteristic (ROC) curves. Receiver operator characteristic (ROC) curves for discriminating between hospital survivors and non-survivors. Values represent those measured on admission to the intensive care unit. APACHE = acute physiology and chronic health evaluation; NLR = neutrophil/lymphocyte ratio; LDH = lactate dehydrogenase.

The APACHE II score had the highest AUC in discriminating non-survivors from survivors (AUC = 0.77; 95% CI: 0.71–0.84, p<0.001), followed by the NLR (AUC = 0.69; 95% CI: 0.61–0.76, p<0.001), and LDH value (AUC = 0.66; 95% CI: 0.59–0.73, p<0.001) (Fig 2). The best cut-off point for the APACHE II score was 15 points with a sensitivity of 61%, specificity of 83%, NPV 89%, and PPV 50%. The best cut-off point for the NLR was 12 with sensitivity of 65%, specificity 59%, NPV 87%, and PPV 45%. LDH levels higher than 462 IU/L discriminated between non-survivors and survivors with a sensitivity of 61%, specificity 66%, NPV 87%, and PPV 31%.

## Discussion

The main findings in this cohort of patients with severe COVID-19 pneumonia admitted to the ICU of a tertiary hospital in the UAE during the first wave of the epidemic were that: 1) patients were most commonly middle age males with preexisting comorbid conditions; 2) COVID-19 was associated with high morbidity and mortality rates; 3) admission from the hospital ward, greater APACHE II score on ICU admission, the occurrence of diarrhea prior to hospital admission, greater NLR, and higher LDH were independently associated with a higher risk of in-hospital mortality; and 4) the APACHE II score had the highest AUC for discriminating non-survivors from survivors, followed by NLR, and LDH.

Patients in our cohort were predominantly middle age males, reflecting the demography of patients who may be at a higher risk of developing severe COVID-19 in our community. Previous large cohorts of COVID-19 patients admitted to the ICU have also shown a high prevalence of male patients [2–5, 17, 18]; however, the mean age reported in these studies was markedly higher than that reported in our study. The relatively high prevalence of preexisting comorbid conditions in our patients may explain in part the increased likelihood of developing severe COVID-19 despite the younger age. Nonetheless, age was not identified as a risk factor for in-hospital death in the multivariable analysis after adjustment for other possible confounders, probably due to the inclusion of APACHE II score in the model, which considers age as an essential component.

Although COVID-19 was associated with a high mortality rate of around 20% in our cohort, this rate was significantly lower than that reported for critically ill COVID-19 patients in previous international cohorts. In a meta-analysis, including literature published on MEDLINE, EMBASE, PubMed, and Cochrane databases up to May 31, 2020, combined ICU mortality was 41.6% [12]. However, most of the studies published to-date have included patients from Asian [10, 15, 21–24], European [2, 3, 5, 9, 11, 14, 16–20], and North American [4, 6, 7, 25] ICUs. Moreover, the healthcare systems in these regions were often overwhelmed in the early months of the epidemic, which may have negatively influenced outcomes in these cohorts. Indeed, the meta-analysis by Armstrong et al. showed that combined ICU mortality in the included studies decreased from 59.5 to 41.6% as the epidemic progressed. We may also speculate that the mortality rate was relatively low in our unit because of the younger age of the patients admitted to our ICU. Indeed, survivors in our study were significantly younger than non-survivors.

We also found that the occurrence of diarrhea prior to hospital admission was independently associated with a high risk of in-hospital death. In a meta-analysis including a total of 4805 patients with COVID-19, 7.4% of the patients had reported diarrhea and a total of 12% had reported gastrointestinal symptoms [31]. The high prevalence of gastrointestinal manifestations may be explained, at least in part, by the high expression of angiotensin-converting enzyme 2 receptors along the epithelial lining of the gut, which act as host-cell receptors for SARS-CoV-2 [32]. An association between SARS-CoV-2 infections and gastrointestinal disorders was confirmed by El Moheb et al. [33], who found a higher rate of gastrointestinal complications in critically ill patients with COVID-19, including mesenteric ischemia, compared with propensity score–matched patients without COVID-19, suggesting a distinct phenotype for COVID-19 compared with conventional acute respiratory distress syndrome (ARDS). Previous studies have also demonstrated that COVID-19-associated digestive symptoms may be associated with poor outcome [34]. In a meta-analysis by Mao et al., patients with gastrointestinal symptoms had an increased risk of ARDS and liver injury; however, the pooled rates of discharge, length of hospital stay, and mortality were similar in patients with and without gastrointestinal symptoms [35]. The lack of routine adjustment for possible confounders in the studies included in this analysis [35] and the marked heterogeneity among the studies may have masked a possible association between gastrointestinal symptoms and mortality.

Patients were either admitted directly to the ICU from our emergency department, from the emergency departments of other hospitals, or from the hospital ward. Admissions from the hospital ward represent, therefore, patients who spend several days in the hospital prior to ICU admission. We may assume that these patients may have been more liable to hospital acquired complications, are at a more advanced stage throughout the disease trajectory, and have already received available therapies without initial success. We may also assume that early intensive care management may be associated with improved outcome in these patients. The admission/discharge policies and abundant availability of ICU resources, together with the local cultural values, have probably lead to systematic referral of severely ill patients to the ICU, irrespective of the possible outcome. This may explain the higher risk of death in patients referred from the hospital ward compared to primary ICU admissions in our cohort.

Although the APACHE II score was relatively low in our cohort, it had the highest AUC for discriminating non-survivors from survivors. The best cut-off point for the APACHE II score was only 15 points. Our findings are in agreement with those of Zou et al. who showed that an APACHE II score with a cut-off point of 17 was a more effective clinical tool to predict hospital mortality in patients with COVID-19 disease than the Sequential Organ Failure Assessment (SOFA) and CURB-65 scores [36]. Low APACHE II scores on admission to the ICU have consistently been reported in COVID-19 patients [9, 11, 13, 23, 25, 36], probably due to the predominance of respiratory failure, with minimal impairment of other physiologic parameters in the early phase of the disease process. In agreement with earlier studies [37, 38], we found that the NLR was a good predictor of mortality in patients with COVID-19. SARS-CoV-2 infection may be associated with both excessive inflammation [39] and immune suppression [40]. Accordingly, the lymphocyte count decreases progressively, whereas the neutrophil count increases in patients with severe COVID-19 [37, 38]. Indeed, a pooled analysis of 10 studies, including 2967 COVID-19 patients, found that an elevated NLR had both sensitivity and specificity of 0.83 for discriminating between survivors and non-survivors [41]. The elevation of NLR in ICU patients compared patients with milder disease forms may be explained by the time lag between the onset of the disease and ICU admission, enabling NLR to reach higher levels than those reported in patients hospitalized with mild disease progression. Elevated LDH levels were independently associated with a higher risk of in-hospital death in our study. LDH is an intracellular enzyme in the glycolytic pathway, which catalyzes the interconversion of pyruvate and lactate [42]. Since LDH is present in lung tissue, patients with severe COVID-19 infection can be expected to release greater amounts of LDH into the circulation [43, 44]. Elevated LDH levels may also be associated with multiple organ dysfunction and subsequently poor outcome [43]. In a pooled analysis of 9 studies including 1532 COVID-19 patients, Henry et al. found that elevated LDH levels, measured at the earliest possible time after hospital admission, were associated with a 6-fold increase in the odds of developing severe disease and a 16-fold increase in the odds of mortality in these patients [43]. The presence of a high LDH value on admission to the ICU may, therefore, be a potential marker of poor prognosis in critically ill patients with COVID-19.

Survivors may be more likely to stay for a short period of time in the ICU as they are less sick than non-survivors and are, hence, more likely to spend more time in the hospital ward after discharge from the ICU. This may explain the shorter ICU and longer hospital LOS in survivors than no-survivors in our cohort. As expected, invasive mechanical ventilation, the need for hemodialysis, and vasopressor therapy were more prevalent in non-survivors than survivors, which reflect the degree of organ dysfunction/failure and its relation to outcome in these patients.

Our study has some limitations including that it was retrospective and the multivariable analysis is limited by the variables included in the analysis, so that the possible effect of unmeasured confounders cannot be excluded. Our cohort may also not be representative of all critically ill patients admitted to ICUs in the UAE during the first wave of the epidemic. Indeed, our patients included different ethnic groups, but were mainly of Asian origin.

## Conclusion

In this cohort of patients admitted to the ICU of a tertiary hospital in the UAE, COVID-19 pneumonia was associated with high morbidity and mortality rates. Admission from the hospital ward, greater APACHE II score on ICU admission, diarrhea prior to hospital admission, greater NLR, and higher LDH value were independently associated with a higher risk of in-hospital mortality.

## Supporting information

S1 Table Anonymized data set of the 371 patients included in the study. Age and ethnicity were masked by the authors to avoid patients’ identification.
(XLS)

S2 Table Characteristics of the two major ethnic groups included in the study. (DOCX)

S3 Table A. Variables retained in the predictive equation throughout the various steps of the multivariable modeling. B. Variable not retained in the predictive equation throughout the various steps of the multivariable modeling. C. Hosmer & Lemeshow goodness of fit test throughout the consecutive steps of the multivariable model.
(DOCX)
