# Supplementary material for: Characteristics and outcome of critically ill patients with coronavirus disease-2019 (COVID-19) pneumonia admitted to a tertiary care center in the United Arab Emirates during the first wave of the SARS-CoV-2 pandemic. A retrospective analysis
Source: PLoS One. 2021 Oct 22;16(10):e0251687. doi: 10.1371/journal.pone.0251687 (PMC8535183; doi:10.1371/journal.pone.0251687)
Supplement: S2 Table — (DOCX) [file pone.0251687.s002.docx]

S2 Table. Characteristics of the two major ethnic groups included in the study

|  | Arab | South Asian | p value |
| --- | --- | --- | --- |
| N | 116 | 231 |  |
| Age, years, mean ±SD | 62±15 | 49±11 | <0.001 |
| Male (%) | 77 (66.4) | 220 (95.2) | <0.001 |
| BMI, kg/m^2^, mean ±SD | 30.8± 7.9 | 32.2± 28.8 | 0.011 |
| Comorbid conditions, n (%) |  |  |  |
| Diabetes mellitus | 68 (58.6) | 88 (38.1) | <0.001 |
| Systemic hypertension | 67 (57.8) | 83 (35.9) | <0.001 |
| Cardiovascular disease, any | 35 (28.4) | 27 (11.7) | <0.001 |
| Ischemic heart diease | 22 (19.0) | 18 (7.8) | 0.002 |
| Congestive heart failure | 5 (4.3) | 5 (2.2) | 0.312 |
| Atrial fibrillation/flutter | 5 (4.3) | 2 (0.9) | 0.044 |
| Heart block | 1 (0.9) | 2 (0.9) | 1.000 |
| Valvular heart disease | 1 (0.9) | - | 0.334 |
| Peripheral vasucular disease | - | 1 (0.4) | 1.000 |
| Chronic renal disease, any | 24 (20.7) | 24 (10.4) | 0.009 |
| End stage renal disease | 4 (3.4) | 2 (0.9) | 0.099 |
| APACHE II score, mean ±SD | 14±9 | 10±7 | <0.001 |

APACHE II: Acute physiologic and chronic health evaluation score. BMI: body mass index. SD: standard deviation.
