# Supplementary material for: Characteristics and outcome of critically ill patients with coronavirus disease-2019 (COVID-19) pneumonia admitted to a tertiary care center in the United Arab Emirates during the first wave of the SARS-CoV-2 pandemic. A retrospective analysis
Source: PLoS One. 2021 Oct 22;16(10):e0251687. doi: 10.1371/journal.pone.0251687 (PMC8535183; doi:10.1371/journal.pone.0251687)
Supplement: S3 Table — A. Variables retained in the predictive equation throughout the various steps of the multivariable modeling. B. Variable not retained in the predictive equation throughout the various steps of the multivariable modeling. C. Hosmer & Lemeshow goodness of fit test throughout the consecutive steps of the multivariable model. (DOCX) [file pone.0251687.s003.docx]

S3 A Table. Variables retained in the predictive equation throughout the various steps of the multivariable modeling*

|  | | Regression coefficient | Standard  error | Wald statistics | Odds ratio | 95% confidence interval | |  |
| --- | --- | --- | --- | --- | --- | --- | --- | --- |
|  |  |  |  |  |  | Lower limit | Upper limit | p value |
| Step 1 | APACHE II score | .129 | .019 | 44.571 | 1.138 | 1.095 | 1.181 | <.0001 |
|  | Constant | -3.028 | .312 | 94.084 | .048 |  |  | <.0001 |
| Step 2 | Neutrophil:lymphocyte ratio | .045 | .017 | 7.127 | 1.046 | 1.012 | 1.081 | .008 |
|  | APACHE II score | .120 | .020 | 36.813 | 1.127 | 1.084 | 1.172 | <.001 |
|  | Constant | -3.357 | .345 | 94.797 | .035 |  |  | <.0001 |
| Step 3 | Lactate dehydrogenase | .002 | .001 | 5.806 | 1.002 | 1.000 | 1.003 | .016 |
|  | Neutrophil:lymphocyte ratio | .043 | .017 | 6.477 | 1.044 | 1.010 | 1.078 | .011 |
|  | APACHE II score | .119 | .020 | 36.257 | 1.127 | 1.084 | 1.171 | <.0001 |
|  | Constant | -4.148 | .498 | 69.411 | .016 |  |  | <.0001 |
| Step 4 | Referring facility  - Primary admission (reference) |  |  | 9.442 |  |  |  | .024 |
|  | - Other hospital. same city | .509 | .583 | .763 | 1.664 | .531 | 5.215 | .383 |
|  | - Other hospital. another city | -.155 | .376 | .170 | .856 | .409 | 1.791 | .680 |
|  | - Hospital ward | 1.366 | .528 | 6.697 | 3.919 | 1.393 | 11.025 | .010 |
|  | Lactate dehydrogenase | .002 | .001 | 6.225 | 1.002 | 1.000 | 1.003 | .013 |
|  | Neutrophil:lymphocyte ratio | .042 | .017 | 6.299 | 1.043 | 1.009 | 1.078 | .012 |
|  | APACHE II score | .124 | .021 | 34.370 | 1.132 | 1.086 | 1.180 | <.0001 |
|  | Constant | -3.940 | .530 | 55.253 | .019 |  |  | <.0001 |
| Step 5 | Referring facility  - Primary admission (reference) |  |  | 9.698 |  |  |  | .021 |
|  | - Other hospital. same city | .590 | .589 | 1.004 | 1.804 | .569 | 5.720 | .316 |
|  | - Other hospital. another city | -.157 | .379 | .171 | .855 | .407 | 1.797 | .679 |
|  | - Hospital ward | 1.365 | .529 | 6.661 | 3.916 | 1.389 | 11.040 | .010 |
|  | Diarrhea | 1.150 | .580 | 3.936 | 3.158 | 1.014 | 9.838 | .047 |
|  | Lactate dehydrogenase | .002 | .001 | 6.943 | 1.002 | 1.000 | 1.003 | .008 |
|  | Neutrophil:lymphocyte ratio | .042 | .017 | 5.835 | 1.043 | 1.008 | 1.079 | .016 |
|  | APACHE II score | .126 | .021 | 34.901 | 1.135 | 1.088 | 1.183 | <.0001 |
|  | Konstante | -3.528 | .558 | 40.018 | .029 |  |  | <.0001 |
| Step 6 | Referring facility  - Primary admission (reference) |  |  | 9.897 |  |  |  | .019 |
|  | - Other hospital. same city | .410 | .615 | .445 | 1.507 | .451 | 5.033 | .505 |
|  | - Other hospital. another city | -.249 | .385 | .420 | .779 | .367 | 1.657 | .517 |
|  | - Hospital ward | 1.345 | .526 | 6.540 | 3.840 | 1.369 | 10.767 | .011 |
|  | Diarrhea | 1.082 | .597 | 3.280 | 2.949 | .915 | 9.508 | .070 |
|  | Bilirubin, direct | .047 | .027 | 3.032 | 1.048 | .994 | 1.105 | .082 |
|  | Lactate dehydrogenase | .002 | .001 | 4.886 | 1.002 | 1.000 | 1.003 | .027 |
|  | Neutrophil:lymphocyte ratio | .038 | .017 | 4.809 | 1.039 | 1.004 | 1.075 | .028 |
|  | APACHE II score | .124 | .022 | 33.100 | 1.132 | 1.085 | 1.181 | <.0001 |
|  | Constant | -3.675 | .573 | 41.065 | .025 |  |  | <.0001 |
| Step 7 | Referring facility  - Primary admission (reference) |  |  | 9.256 |  |  |  | .026 |
|  | - Other hospital. same city | .446 | .610 | .534 | 1.561 | .473 | 5.156 | .465 |
|  | - Other hospital. another city | -.269 | .389 | .476 | .764 | .356 | 1.640 | .490 |
|  | - Hospital ward | 1.268 | .529 | 5.740 | 3.554 | 1.259 | 10.030 | .017 |
|  | Nausea/Vomiting | -1.317 | .945 | 1.942 | .268 | .042 | 1.708 | .163 |
|  | Diarrhea | 1.311 | .623 | 4.431 | 3.709 | 1.095 | 12.566 | .035 |
|  | Bilirubin, direct | .046 | .027 | 2.794 | 1.047 | .992 | 1.104 | .095 |
|  | Lactate dehydrogenase | .002 | .001 | 4.986 | 1.002 | 1.000 | 1.003 | .026 |
|  | Neutrophil:lymphocyte ratio | .042 | .018 | 5.735 | 1.043 | 1.008 | 1.179 | .017 |
|  | APACHE II score | .123 | .022 | 32.510 | 1.131 | 1.084 | 1.180 | <.0001 |
|  | Constant | -3.551 | .579 | 37.633 | .029 |  |  | <.0001 |

APACHE: acute physiology and chronic health evaluation, CI: Confidence interval,ICU: intensive care unit, OR: odds ratio.

* Forward stepwise analysis with in-hospital death as the dependent variable. Variables were entered in the model at a level of 0.2 and excluded. at 0.1.

S3 B Table. Variable not retained in the predictive equation throughout the various steps of the multivariable modeling

|  |  | Estimate | degree of freedom | p value |
| --- | --- | --- | --- | --- |
| Step 1 | Referring facility  - Primary admission (reference) | 10.058 | 3 | .018 |
|  | - Other hospital. same city | .847 | 1 | .357 |
|  | - Other hospital. another city | 1.994 | 1 | .158 |
|  | - Hospital ward | 8.640 | 1 | .003 |
|  | Ethnicity  - Arab (reference) | 3.467 | 3 | .325 |
|  | - Asian. other | 2.610 | 1 | .106 |
|  | - Others | .586 | 1 | .444 |
|  | - West Asian | .011 | 1 | .916 |
|  | Mechanical ventilation. on ICU admission | .186 | 1 | .666 |
|  | Fever | .426 | 1 | .514 |
|  | Nausea/vomiting | 1.112 | 1 | .292 |
|  | Diarrhea | 3.228 | 1 | .072 |
|  | Malaise | .006 | 1 | .941 |
|  | Wheeze | .155 | 1 | .694 |
|  | Cough | .031 | 1 | .861 |
|  | Productive cough | 1.635 | 1 | .201 |
|  | Dyspnea | .232 | 1 | .630 |
|  | Headache | .177 | 1 | .674 |
|  | Age | .005 | 1 | .941 |
|  | Diabetes mellitus | 1.878 | 1 | .171 |
|  | Systemic hypertension | 1.653 | 1 | .199 |
|  | Ischemic heart disease | .137 | 1 | .712 |
|  | Chronic renal disease. any | .028 | 1 | .866 |
|  | End stage renal disease | .045 | 1 | .832 |
|  | Cardiovascular disease. any | .009 | 1 | .923 |
|  | Creatinine | .270 | 1 | .603 |
|  | Urea | .932 | 1 | .334 |
|  | Bilirubin. direct | 7.416 | 1 | .006 |
|  | C-reactive protein | 3.601 | 1 | .058 |
|  | D-dimer | 4.985 | 1 | .026 |
|  | White blood cell count | .945 | 1 | .331 |
|  | Hemoglobin concentration | .079 | 1 | .779 |
|  | Procalcitonin | .333 | 1 | .564 |
|  | Lactate dehydrogenase | 7.029 | 1 | .008 |
|  | Ferritin | 3.400 | 1 | .065 |
|  | Platelet count | 1.405 | 1 | .236 |
|  | Neutrophil:lymphocyte ratio | 8.258 | 1 | .004 |
|  | Alkaline phosphatase | 1.559 | 1 | .212 |
|  | ALT | .208 | 1 | .648 |
|  | Amylase | .002 | 1 | .966 |
|  | AST | .044 | 1 | .835 |
|  | Lipase | .152 | 1 | .697 |
|  | Albumin | 4.042 | 1 | .044 |
| Step 2 | Referring facility  - Primary admission (reference) | 9.746 | 3 | .021 |
|  | - Other hospital. same city | 1.019 | 1 | .313 |
|  | - Other hospital. another city | 1.757 | 1 | .185 |
|  | - Hospital ward | 8.149 | 1 | .004 |
|  | Ethnicity  - Arab (reference) | 6.053 | 3 | .109 |
|  | - Asian. other | 4.065 | 1 | .044 |
|  | - Others | 1.191 | 1 | .275 |
|  | - West Asian | .004 | 1 | .951 |
|  | Mechanical ventilation. on ICU admission | .088 | 1 | .767 |
|  | Fever | .604 | 1 | .437 |
|  | Nausea/vomiting | 1.604 | 1 | .205 |
|  | Diarrhea | 2.945 | 1 | .086 |
|  | Malaise | .029 | 1 | .864 |
|  | Wheeze | .172 | 1 | .679 |
|  | Cough | .062 | 1 | .803 |
|  | Productive cough | 1.450 | 1 | .228 |
|  | Dyspnea | .065 | 1 | .798 |
|  | Headache | .088 | 1 | .766 |
|  | Age | .006 | 1 | .938 |
|  | Diabetes mellitus | .952 | 1 | .329 |
|  | Systemic hypertension | 1.089 | 1 | .297 |
|  | Ischemic heart disease | .156 | 1 | .693 |
|  | Chronic renal disease. any | .006 | 1 | .941 |
|  | End stage renal disease | .189 | 1 | .664 |
|  | Cardiovascular disease. any | .014 | 1 | .904 |
|  | Creatinine | .372 | 1 | .542 |
|  | Urea | .195 | 1 | .659 |
|  | Bilirubin. direct | 5.725 | 1 | .017 |
|  | C-reactive protein | .981 | 1 | .322 |
|  | D-dimer | 3.424 | 1 | .064 |
|  | White blood cell count | .171 | 1 | .679 |
|  | Hemoglobin concentration | .262 | 1 | .609 |
|  | Procalcitonin | .065 | 1 | .799 |
|  | Lactate dehydrogenase | 6.052 | 1 | .014 |
|  | Ferritin | 2.524 | 1 | .112 |
|  | Platelet count | 1.536 | 1 | .215 |
|  | Alkaline phosphatase | .587 | 1 | .444 |
|  | ALT | .082 | 1 | .774 |
|  | Amylase | .039 | 1 | .843 |
|  | AST | .011 | 1 | .916 |
|  | Lipase | .152 | 1 | .697 |
|  | Albumin | 2.003 | 1 | .157 |
| Step 3 | Referring facility  - Primary admission (reference) | 10.224 | 3 | .017 |
|  | - Other hospital. same city | .750 | 1 | .386 |
|  | - Other hospital. another city | 2.400 | 1 | .121 |
|  | - Hospital ward | 8.850 | 1 | .003 |
|  | Ethnicity  - Arab (reference) | 5.167 | 3 | .160 |
|  | - Asian. other | 3.981 | 1 | .046 |
|  | - Others | .911 | 1 | .340 |
|  | - West Asian | .161 | 1 | .688 |
|  | Mechanical ventilation. on ICU admission | .062 | 1 | .803 |
|  | Fever | .693 | 1 | .405 |
|  | Nausea/vomiting | 1.650 | 1 | .199 |
|  | Diarrhea | 3.822 | 1 | .051 |
|  | Malaise | .019 | 1 | .889 |
|  | Wheeze | .253 | 1 | .615 |
|  | Cough | .115 | 1 | .734 |
|  | Productive cough | .813 | 1 | .367 |
|  | Dyspnea | .239 | 1 | .625 |
|  | Headache | .113 | 1 | .737 |
|  | Age | .372 | 1 | .542 |
|  | Diabetes mellitus | .374 | 1 | .541 |
|  | Systemic hypertension | .311 | 1 | .577 |
|  | Ischemic heart disease | .388 | 1 | .533 |
|  | Chronic renal disease. any | .163 | 1 | .687 |
|  | End stage renal disease | .294 | 1 | .587 |
|  | Cardiovascular disease. any | .193 | 1 | .661 |
|  | Creatinine | .623 | 1 | .430 |
|  | Urea | .284 | 1 | .594 |
|  | Bilirubin. direct | 3.552 | 1 | .059 |
|  | C-reactive protein | 1.076 | 1 | .300 |
|  | D-dimer | 1.320 | 1 | .251 |
|  | White blood cell count | .813 | 1 | .367 |
|  | Hemoglobin concentration | .285 | 1 | .593 |
|  | Procalcitonin | .092 | 1 | .762 |
|  | Ferritin | .495 | 1 | .482 |
|  | Platelet count | 1.378 | 1 | .240 |
|  | Alkaline phosphatase | .420 | 1 | .517 |
|  | ALT | .032 | 1 | .859 |
|  | Amylase | .416 | 1 | .519 |
|  | AST | .484 | 1 | .487 |
|  | Lipase | 1.051 | 1 | .305 |
|  | Albumin | 1.090 | 1 | .297 |
| Step 4 | Ethnicity  - Arab (reference) | 6.739 | 3 | .081 |
|  | - Asian. other | 4.821 | 1 | .028 |
|  | - Others | 1.041 | 1 | .308 |
|  | - West Asian | .011 | 1 | .916 |
|  | Mechanical ventilation. on ICU admission | .101 | 1 | .751 |
|  | Fever | 1.208 | 1 | .272 |
|  | Nausea/vomiting | 1.294 | 1 | .255 |
|  | Diarrhea | 4.140 | 1 | .042 |
|  | Malaise | .048 | 1 | .826 |
|  | Wheeze | .226 | 1 | .634 |
|  | Cough | .702 | 1 | .402 |
|  | Productive cough | .829 | 1 | .363 |
|  | Dyspnea | .001 | 1 | .981 |
|  | Headache | .259 | 1 | .611 |
|  | Age | .256 | 1 | .613 |
|  | Diabetes mellitus | .649 | 1 | .420 |
|  | Systemic hypertension | .565 | 1 | .452 |
|  | Ischemic heart disease | .415 | 1 | .519 |
|  | Chronic renal disease. any | .418 | 1 | .518 |
|  | End stage renal disease | .607 | 1 | .436 |
|  | Cardiovascular disease. any | .092 | 1 | .762 |
|  | Creatinine | 1.072 | 1 | .300 |
|  | Urea | .529 | 1 | .467 |
|  | Bilirubin. direct | 3.856 | 1 | .050 |
|  | C-reactive protein | 1.379 | 1 | .240 |
|  | D-dimer | 1.598 | 1 | .206 |
|  | White blood cell count | .337 | 1 | .562 |
|  | Hemoglobin concentration | .054 | 1 | .817 |
|  | Procalcitonin | .042 | 1 | .838 |
|  | Ferritin | .399 | 1 | .528 |
|  | Platelet count | 1.219 | 1 | .270 |
|  | Alkaline phosphatase | .737 | 1 | .391 |
|  | ALT | .032 | 1 | .858 |
|  | Amylase | .497 | 1 | .481 |
|  | AST | .411 | 1 | .521 |
|  | Lipase | 1.246 | 1 | .264 |
|  | Albumin | .949 | 1 | .330 |
| Step 5 | Ethnicity  - Arab (reference) | 6.168 | 3 | .104 |
|  | - Asian. other | 4.520 | 1 | .034 |
|  | - Others | .985 | 1 | .321 |
|  | - West Asian | .050 | 1 | .823 |
|  | Mechanical ventilation. on ICU admission | .124 | 1 | .725 |
|  | Fever | 1.325 | 1 | .250 |
|  | Nausea/vomiting | 2.243 | 1 | .134 |
|  | Malaise | .178 | 1 | .673 |
|  | Wheeze | .211 | 1 | .646 |
|  | Cough | .588 | 1 | .443 |
|  | Productive cough | .895 | 1 | .344 |
|  | Dyspnea | .001 | 1 | .973 |
|  | Headache | .374 | 1 | .541 |
|  | Age | .340 | 1 | .560 |
|  | Diabetes mellitus | .559 | 1 | .455 |
|  | Systemic hypertension | .450 | 1 | .503 |
|  | Ischemic heart disease | .532 | 1 | .466 |
|  | Chronic renal disease. any | .479 | 1 | .489 |
|  | End stage renal disease | .684 | 1 | .408 |
|  | Cardiovascular disease. any | .187 | 1 | .665 |
|  | Creatinine | 1.269 | 1 | .260 |
|  | Urea | .665 | 1 | .415 |
|  | Bilirubin. direct | 3.114 | 1 | .078 |
|  | C-reactive protein | 1.641 | 1 | .200 |
|  | D-dimer | 1.696 | 1 | .193 |
|  | White blood cell count | .404 | 1 | .525 |
|  | Hemoglobin concentration | .184 | 1 | .668 |
|  | Procalcitonin | .068 | 1 | .794 |
|  | Ferritin | .553 | 1 | .457 |
|  | Platelet count | 1.205 | 1 | .272 |
|  | Alkaline phosphatase | .779 | 1 | .378 |
|  | ALT | .046 | 1 | .829 |
|  | Amylase | .411 | 1 | .522 |
|  | AST | .544 | 1 | .461 |
|  | Lipase | 1.126 | 1 | .289 |
|  | Albumin | .676 | 1 | .411 |
| Step 6 | Ethnicity  - Arab (reference) | 5.960 | 3 | .114 |
|  | - Asian. other | 4.422 | 1 | .035 |
|  | - Others | .796 | 1 | .372 |
|  | - West Asian | .014 | 1 | .904 |
|  | Mechanical ventilation. on ICU admission | .275 | 1 | .600 |
|  | Fever | .853 | 1 | .356 |
|  | Nausea/vomiting | 2.029 | 1 | .154 |
|  | Malaise | .147 | 1 | .701 |
|  | Wheeze | .204 | 1 | .652 |
|  | Cough | .217 | 1 | .642 |
|  | Productive cough | 1.075 | 1 | .300 |
|  | Dyspnea | .020 | 1 | .887 |
|  | Headache | .314 | 1 | .575 |
|  | Age | .555 | 1 | .456 |
|  | Diabetes mellitus | .342 | 1 | .559 |
|  | Systemic hypertension | .349 | 1 | .554 |
|  | Ischemic heart disease | .515 | 1 | .473 |
|  | Chronic renal disease. any | .448 | 1 | .503 |
|  | End stage renal disease | .939 | 1 | .333 |
|  | Cardiovascular disease. any | .253 | 1 | .615 |
|  | Creatinine | 1.563 | 1 | .211 |
|  | Urea | .495 | 1 | .482 |
|  | C-reactive protein | 1.348 | 1 | .246 |
|  | D-dimer | 1.122 | 1 | .289 |
|  | White blood cell count | .633 | 1 | .426 |
|  | Hemoglobin concentration | .207 | 1 | 649 |
|  | Procalcitonin | .097 | 1 | .755 |
|  | Ferritin | .456 | 1 | .499 |
|  | Platelet count | .817 | 1 | .366 |
|  | Alkaline phosphatase | .313 | 1 | .576 |
|  | ALT | .191 | 1 | .662 |
|  | Amylase | .470 | 1 | .493 |
|  | AST | .881 | 1 | .348 |
|  | Lipase | 1.527 | 1 | .216 |
|  | Albumin | .380 | 1 | .538 |
| Step 7 | Ethnicity  - Arab (reference) | 5.167 | 3 | .160 |
|  | - Asian. other | 3.981 | 1 | .046 |
|  | - Others | .911 | 1 | .340 |
|  | - West Asian | .161 | 1 | .688 |
|  | Mechanical ventilation. on ICU admission | .342 | 1 | .559 |
|  | Fever | .878 | 1 | .349 |
|  | Malaise | .184 | 1 | .668 |
|  | Wheeze | .218 | 1 | .641 |
|  | Cough | .104 | 1 | .747 |
|  | Productive cough | 1.516 | 1 | .218 |
|  | Dyspnea | .017 | 1 | .896 |
|  | Headache | .334 | 1 | .563 |
|  | Age | .498 | 1 | .480 |
|  | Diabetes mellitus | .207 | 1 | .649 |
|  | Systemic hypertension | .360 | 1 | .549 |
|  | Ischemic heart disease | .538 | 1 | .463 |
|  | Chronic renal disease. any | .461 | 1 | .497 |
|  | End stage renal disease | 1.427 | 1 | .232 |
|  | Cardiovascular disease. any | .215 | 1 | .643 |
|  | Creatinine | 2.180 | 1 | .140 |
|  | Urea | .427 | 1 | .514 |
|  | C-reactive protein | 1.234 | 1 | .267 |
|  | D-dimer | 1.300 | 1 | .254 |
|  | White blood cell count | .999 | 1 | .317 |
|  | Hemoglobin concentration | .043 | 1 | .836 |
|  | Procalcitonin | .392 | 1 | .531 |
|  | Ferritin | .328 | 1 | .567 |
|  | Platelet count | .912 | 1 | .340 |
|  | Alkaline phosphatase | .187 | 1 | .665 |
|  | ALT | .076 | 1 | .783 |
|  | Amylase | .535 | 1 | .464 |
|  | AST | .740 | 1 | .390 |
|  | Lipase | 1.558 | 1 | .212 |
|  | Albumin | .239 | 1 | .625 |

ALT: alanine aminotransferase. AST: aspartate transaminase, ICU: intensive care unit

S3 C Hosmer & Lemeshow goodness of fit test throughout the consecutive steps of the multivariable model

| Step | Chi square | degree of freedom | p value |
| --- | --- | --- | --- |
| 1 | 6.920 | 8 | .545 |
| 2 | 3.806 | 8 | .874 |
| 3 | 4.792 | 8 | .780 |
| 4 | 14.165 | 8 | .078 |
| 5 | 7.992 | 8 | .434 |
| 6 | 6.790 | 8 | .559 |
| 7 | 7.364 | 8 | .460 |
